# Supplementary material for: Identification of photoperiod-induced specific miRNAs in the adrenal glands of Sunite sheep (Ovis aries)
Source: Front Vet Sci. 2022 Jul 22;9:888207. doi: 10.3389/fvets.2022.888207 (PMC9354845; doi:10.3389/fvets.2022.888207)
Supplement: Supplementary file 1 [file Data_Sheet_1.ZIP › Supplementary file/miRDeep2 file.zip!/miRDeep2 file/Novel_Structure/LP42A1/Novel_240.pdf]

Provisional ID	:	NC_019484.2_35565
Score total	:	1.8
Score for star read(s)	:	-1.3
Score for read counts	:	0
Score for mfe	:	1.5
Score for randfold	:	1.6
Score for cons. seed	:	
Total read count	:	72
Mature read count	:	72
Loop read count	:	0
Star read count	:	0

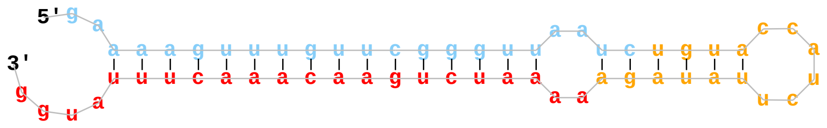

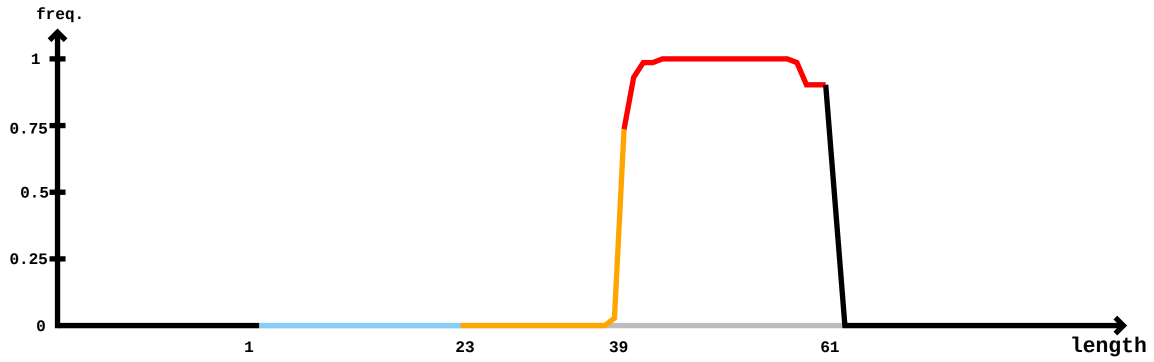

Star   Mature

```
5'- acaauccuagguguuggcugaaaaguuuguucggguuaaaucuguaccaucuuauagaaaaaucugaacaaacuuuauggcuaacccaguauuuucucuucuuaccugaa   -3'    exp
    .........((.((((((((.((((((((((((((((..((((((......))))))..)))))))))))))))).))))))))))........................    reads      mm         sample
    .........................................................aaaaaCcugaacaaacuuua.................................    2          1          seq
    ..........................................................aaaaucugaacaaaUuuu..................................    1          1          seq
    ..........................................................aaaaucugaacaaacuuuU.................................    4          1          seq
    ..........................................................aaaaucugaacaaacuuuUugg..............................    45         1          seq
    ..........................................................aaaaucugaacaaacuuuUuggc.............................    1          1          seq
    ...........................................................aaaucugaacaaacuuuUugg..............................    14         1          seq
    ............................................................aaucugaacaaacuuuUuggc.............................    4          1          seq
    ..............................................................ucugaacaaacuuuUuggc.............................    1          1          seq
```
